# Sensitivity, Specificity, and Predictive Values: Foundations, Pliabilities, and Pitfalls in Research and Practice

**DOI:** 10.3389/fpubh.2017.00307

**Published:** 2017-11-20

**Authors:** Robert Trevethan

**Affiliations:** ^1^Independent academic researcher and author, Albury, NSW, Australia

**Keywords:** screening tests, sensitivity, specificity, positive predictive values, negative predictive values, clinical decision making

## Abstract

Within the context of screening tests, it is important to avoid misconceptions about sensitivity, specificity, and predictive values. In this article, therefore, foundations are first established concerning these metrics along with the first of several aspects of pliability that should be recognized in relation to those metrics. Clarification is then provided about the definitions of sensitivity, specificity, and predictive values and why researchers and clinicians can misunderstand and misrepresent them. Arguments are made that sensitivity and specificity should usually be applied only in the context of describing a screening test’s attributes relative to a reference standard; that predictive values are more appropriate and informative in actual screening contexts, but that sensitivity and specificity can be used for screening decisions about individual people if they are extremely high; that predictive values need not always be high and might be used to advantage by adjusting the sensitivity and specificity of screening tests; that, in screening contexts, researchers should provide information about all four metrics and how they were derived; and that, where necessary, consumers of health research should have the skills to interpret those metrics effectively for maximum benefit to clients and the healthcare system.

## Introduction

There are arguably two kinds of tests used for assessing people’s health: diagnostic tests and screening tests. Diagnostic tests are regarded as providing definitive information about the presence or absence of a target disease or condition. By contrast, screening tests—which are the focus of this article—typically have advantages over diagnostic tests such as placing fewer demands on the healthcare system and being more accessible as well as less invasive, less dangerous, less expensive, less time-consuming, and less physically and psychologically discomforting for clients. Screening tests are also, however, well-known for being imperfect and they are sometimes ambiguous. It is, therefore, important to determine the extent to which these tests are able to identify the likely presence or absence of a condition of interest so that their findings encourage appropriate decision making.

If practitioners are confident when using screening tests, but their confidence is not justified, the consequences could be serious for both individuals and the healthcare system (1, 2). It is important, therefore, that confusion should be avoided with regard to how the adequacy and usefulness of screening tests are determined and described. In this article, an attempt is made to identify why confusion can exist, how it might be resolved, and how, once resolved, improvements could be made with regard to the description and use of screening tests. The focus is on the sensitivity, specificity, and predictive values of those tests.

## Determining Sensitivity, Specificity, and Predictive Values

**Figure 1:**
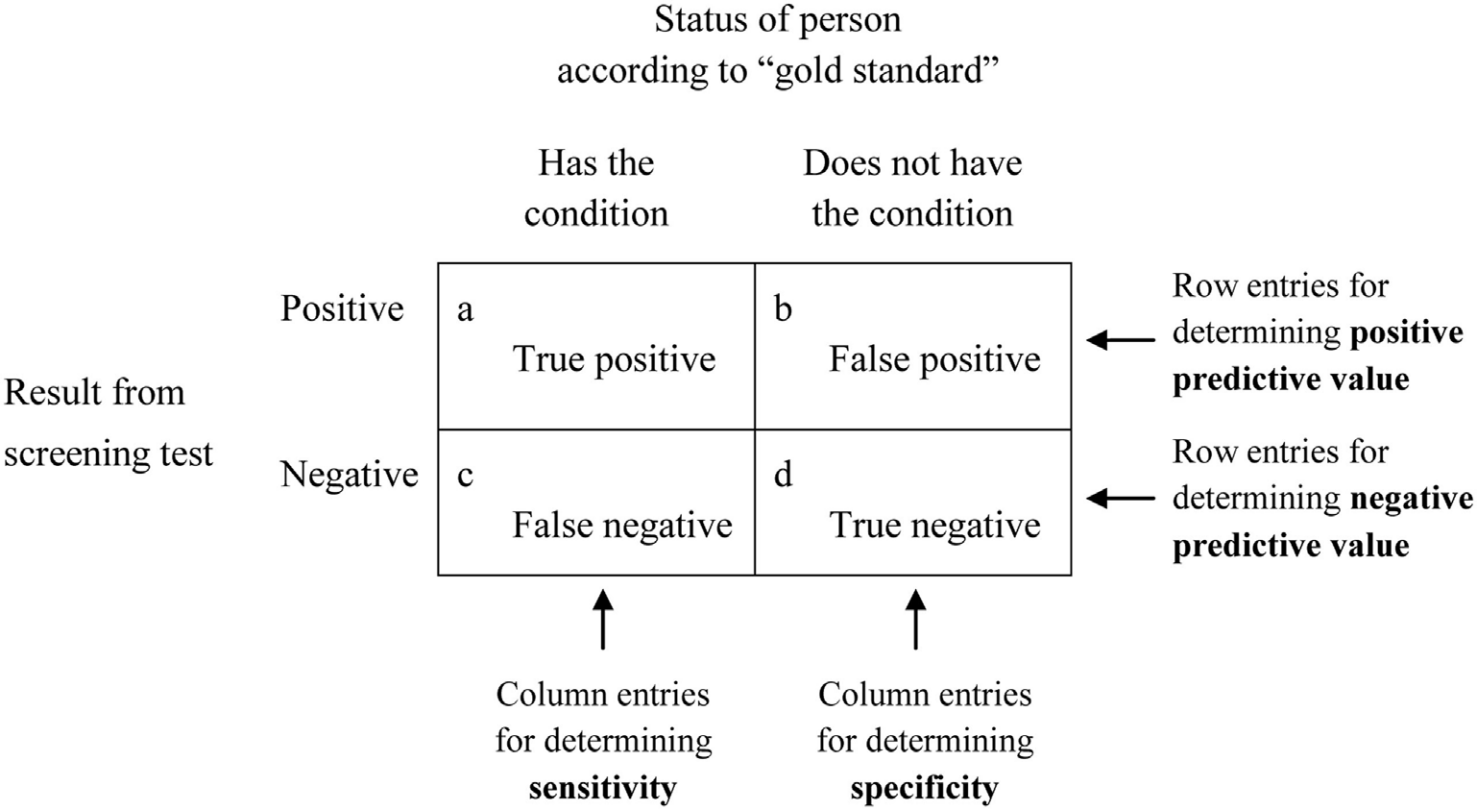
Diagram demonstrating the basis for deriving sensitivity, specificity, and positive and negative predictive values.

When the adequacy, also known as the predictive power or predictive validity, of a screening test is being established, the outcomes yielded by that screening test are initially inspected to see whether they correspond to what is regarded as a definitive indicator, often referred to as a gold standard, of the same target condition. The analyses are typically characterized in the way shown in Figure 1. There it can be seen from the two columns under the heading *Status of person according to “gold standard”* that people are categorized as either having, or as not having, the target condition. The words “gold standard” suggest that this initial categorization is made on the basis of a test that provides authoritative, and presumably indisputable, evidence that a condition does or does not exist. Because there can be concerns about the validity of these so-called gold standards (3, 4), they have increasingly been referred to less glowingly as reference standards (5), thus removing what seemed to be unreserved endorsement. That wording (i.e., reference standard) will be used for the remainder of this article.

Independent of the categorization established on the basis of the reference standard, people are also assessed on the screening test of interest. That test might comprise a natural dichotomy or it might be based on whether the test outcomes fall below or above a specified cutoff point on a continuum. It might also comprise a battery of tests that, together, are regarded as a single test (6–8).

Based on their reference standard and screening test results, people are assigned to one of the four cells labeled a through d in Figure 1 depending on whether they are definitely regarded as having or as not having the target condition based on the reference standard, and whether the screening test yielded a positive result (the person *appears* to have the condition) or a negative result (the person appears *not* to have the condition). What are referred to as sensitivity, specificity, and predictive values can then be calculated from the numbers of people in each of the four cells, and, if expressed as percentages, are based on the following formulas:

$$\begin{array}{l} \text{Sensitivity}=[\text{a}/(\text{a}+\text{c})]\times 100\\ \text{Specificity}=[\text{d}/(\text{b}+\text{d})]\times 100\\ \text{Positive predictive value}\left(\text{PPV}\right)=[\text{a}/(\text{a}+\text{b})]\times 100\\ \text{Negative predictive value}\left(\text{NPV}\right)=[\text{d}/(\text{c}+\text{d})]\times 100. \end{array}$$

These are the metrics that are cited—i.e., often as percentages, although sometimes as decimal fractions, and preferably with accompanying 95% confidence intervals—when researchers and clinicians refer to sensitivity, specificity, and predictive values to describe the characteristics of a screening test. The simplicity, and even familiarity, of these four metrics can mask the existence of a number of complexities that sometimes appear to be underappreciated, however. Deficiencies in either the reference standard or the screening test, or in both, can exist. Furthermore, the four metrics should not be regarded as unquestionably valid and fixed attributes of a screening test: the values that are entered into the cells of Figure 1 depend on how stringent the screening test is and the prevalence of the target condition in the sample of people used in the analysis.

Because of these complexities, it is sometimes necessary to examine the validity of measurement procedures within both the reference standard and the screening test (3, 8). It might also be necessary to question the stringency of the screening test and to ensure that there is a match between the samples that were used for assessing a screening test and the people subsequently being screened (2, 3, 9–11).

It is also important to recognize that there are sometimes noticeable tradeoffs between sensitivity and specificity, as well as between positive predictive values (PPVs) and negative predictive values (NPVs). This is demonstrated in the first four rows of entries in Table 1. Furthermore, as also illustrated in Table 1, there is little or no consistency regarding either size or pattern of sensitivity, specificity, and predictive values in different contexts, so it is not possible to determine one of them merely from information about any of the others. In that sense, they are pliable in relation to each other. This indicates that it is necessary to appreciate the foundations of, distinctions between, and uses and misuses of each of these metrics, and that it is necessary to provide information about all of them, as well as the reference standard and the sample on which they are based, to characterize a screening test adequately.

**Table 1:** Five sets of sensitivity, specificity, and predictive values demonstrating differing patterns of results.

Research domain/researchers	Sensitivity (%)	Specificity (%)	PPV (%)	NPV (%)
Shoulder pain (6)	96	7	15	90
Carpal tunnel syndrome (12)	5	98	10	96
Peripheral artery disease (13)	45	100	100	53
Aspiration risk following stroke (14)	47	86	50	85
Peripheral artery disease (15)	71	79	72	77

*PPV, positive predictive value; NPV, negative predictive value*.

Because sensitivity seems often to be confused with PPV, and specificity seems often to be confused with NPV, unambiguous definitions for each pair are necessary. These are provided below.

## Definitions

### Defining Sensitivity and PPV

The sensitivity of a screening test can be described in variety of ways, typically such as sensitivity being the ability of a screening test to detect a true positive, being based on the true positive rate, reflecting a test’s ability to correctly identify all people who have a condition, or, if 100%, identifying all people with a condition of interest by those people testing positive on the test.

Each of these definitions is incontestably accurate, but they can all be easily misinterpreted because none of them sufficiently emphasizes an important distinction between two essentially different contexts. In the first context, only those people who obtain positive results on the reference standard are assessed in terms of whether they obtained positive or negative results on the screening test. This determines the test’s sensitivity. In the second context, the focus changes from people who tested positive on the *reference standard* to people who tested positive on the *screening test*. Here, an attempt is made to establish whether people who tested positive on the screening test do or do not actually have the condition of interest. This refers to the screening test’s PPV. Expressed differently, the first context is the screening test being assessed on the basis of its performance relative to a reference standard, which focuses on whether the *foundations* of the screening test are satisfactory; the second context is people being assessed on the basis of a screening test, which focuses on the practical *usefulness* of the test in clinical practice.

By way of further explanation, sensitivity is based solely on the cells labeled a and c in Figure 1 and, therefore, requires that all people in the analysis are diagnosed according to the reference standard as definitely having the target condition. The determination of sensitivity does not take into account any people who, according to the reference standard, do not have the condition of interest (who are in cells b and d). Confidence in a screening test’s ability, when it returns a positive result, to differentiate successfully between people who have a condition and those who do not, is another matter. As indicated above, it is the test’s PPV, and is based on the cells labeled a and b, which refer solely to the accuracy of positive results produced by the screening test. Those cells do not include any people who, according to results from the screening test, do not have the condition (who are in cells c and d).

Therefore, a clear definition of sensitivity—with italics for supportive emphasis—would be a screening test’s probability of correctly identifying, *solely from among people who are known to have a condition*, all those who do indeed have that condition (i.e., identifying true positives), and, at the same time, *not* categorizing other people as not having the condition when in fact they *do* have it (i.e., avoiding false negatives). Less elaborated, but perhaps also less helpfully explicit, definitions are possible, for example, that sensitivity is the proportion of people with a condition who are correctly identified by a screening test as indeed having that condition.

It follows that a clear definition of PPV would be a screening test’s probability, when returning a positive result, of correctly identifying, *from among people who might or might not have a condition, all people who do actually have that condition* (i.e., identifying true positives), and, at the same time, not categorizing some people as having the condition when in fact they do not (i.e., avoiding false positives). Expressed differently and more economically, PPV is the probability that people with a positive screening test result indeed do have the condition of interest.

Inspection of Figure 1 supports the above definitions and those that are provided within the next subsection.

### Defining Specificity and NPV

The specificity of a test is defined in a variety of ways, typically such as specificity being the ability of a screening test to detect a true negative, being based on the true negative rate, correctly identifying people who do not have a condition, or, if 100%, identifying all patients who do not have the condition of interest by those people testing negative on the test.

As with the definitions often offered for sensitivity, these definitions are accurate but can easily be misinterpreted because they do not sufficiently indicate the distinction between two different contexts that parallel those identified for sensitivity. Specificity is based on the cells labeled b and d in Figure 1 and, therefore, requires that all the people in the analysis are diagnosed, according to a reference standard, as *not* having the target condition. Specificity does not take into account any people who, according to the reference standard, *do* have the condition (as pointed out above, those people, in the cells labeled a and c, were taken into account when determining sensitivity). Confidence in a screening test’s ability, when it returns a negative result, to differentiate between people who have a condition and those who do not, is another matter. That is the test’s NPV and is based on the cells labeled c and d, which refer solely to the accuracy of negative results produced by the screening test. Those cells do not include any people who, according to the screening test, do have the condition (who are located in cells a and b).

Therefore, a clear definition of specificity, again with italics for supportive emphasis, would be a screening test’s probability of correctly identifying, *solely from among people who are known not to have a condition*, all those who do indeed not have that condition (i.e., identifying true negatives), and, at the same time, *not* categorizing some people as having the condition when in fact they do not have it (i.e., avoiding false positives). Less elaborated, but perhaps also less helpfully explicit, definitions are possible, for example, that specificity is the proportion of people without a condition who are correctly identified by a screening test as indeed not having the condition.

It follows that a clear definition of NPV would be a screening test’s probability, when returning a negative result, of correctly identifying, *from among people who might or might not have a condition, all people who indeed do not have that condition* (i.e., identifying true negatives), and, at the same time, not categorizing some people as not having the condition when in fact they do (i.e., avoiding false negatives). Expressed differently and more economically, NPV is the probability that people with a negative screening test result indeed do not have the condition of interest.

### Summary Regarding Definitions

Sensitivity and specificity are concerned with the accuracy of a screening test relative to a reference standard. The focus is the *adequacy of the screening test*, or its fundamental “credentials.” The main question is: do the results on the screening test correspond to the results on the reference standard? Here, the screening test is being assessed. By contrast, for PPV and NPV, *people* are being assessed. There are two main questions of relevance in that second situation. First, if a person’s screening test yields a positive result, what is the probability that that person has the relevant condition (PPV)? Second, if the screening test yields a negative result, what is the probability that the person does not have the condition (NPV)?

In order to sharpen the distinction, it could be said that sensitivity and specificity indicate the effectiveness of a test with respect to a trusted “outside” referent, while PPV and NPV indicate the effectiveness of a test for categorizing people as having or not having a target condition. More precisely, sensitivity and specificity indicate the concordance of a test with respect to a chosen referent, while PPV and NPV, respectively, indicate the likelihood that a test can successfully identify whether people do or do not have a target condition, based on their test results.

The two contexts (i.e., the context that relates to sensitivity and specificity, versus the context that relates to the two predictive values) should not be confused with each other. Of particular importance, although it is desirable to have tests with high sensitivity and specificity, the values for those two metrics should not be relied on when making decisions about individual people in screening situations. In that second context, use of PPVs and NPVs is more appropriate. The lack of correspondence between sensitivity, specificity, and predictive values is illustrated by the inconsistent pattern of entries in Table 1 and should become more obvious in the next section.

## Uses and Misuses of Sensitivity and Specificity

Because the pairs of categories into which people are placed when sensitivity and specificity values are calculated are not the same as the pairs of categories that pertain in a screening context, there are not only important distinctions between sensitivity and PPV, and between specificity and NPV, but there are also distinct limitations on sensitivity and specificity for screening purposes. Akobeng [(9), p. 340] has gone so far as to write that “both sensitivity and specificity … are of no practical use when it comes to helping the clinician estimate the probability of disease in individual patients.”

Sensitivity does not provide the basis for informed decisions following positive screening test results because those positive test results could contain many false positive outcomes that appear in the cell labeled b in Figure 1. Those outcomes are ignored in determining sensitivity (cells a and c are used for determining sensitivity). Therefore, *of itself* a positive result on a screening test, even if that test has high sensitivity, is not at all useful for definitely regarding a condition as being present in a particular person. Conversely, specificity does not provide an accurate indication about a negative screening test result because negative outcomes from a screening test could contain many false negative results that appear in the cell labeled c, which are ignored in determining specificity (cells b and d are used for determining specificity). Therefore, *of itself*, a negative result on a screening test with high specificity is not at all useful for definitely ruling out disease in a particular person.

Failing to appreciate the above major constraints on sensitivity and specificity arises from what is known in formal logic as confusion of the inverse (16). An example of this with regard to sensitivity, consciously chosen in a form that makes the problem clear, would be converting the logical proposition *This animal is a dog; therefore it is likely to have four legs* into the illogical proposition *This animal has four legs; therefore it is likely to be a dog*. A parallel confusion of the inverse can occur with specificity. An example of this would be converting the logical proposition *This person is not a young adult; therefore this person is not likely to be a university undergraduate* into the illogical proposition *This person is not a university undergraduate; therefore this person is not likely to be a young adult*.

These examples demonstrate the flaws in believing that a positive result on a highly sensitive test indicates the presence of a condition and that a negative result on a highly specific test indicates the absence of a condition. Instead, it should be emphasized that a highly sensitive test, when yielding a positive result, by no means indicates that a condition is present (many animals with four legs are not dogs), and a highly specific test, when yielding a negative result, by no means indicates that a condition is absent (many young people are not university undergraduates).

Despite the above reservations concerning sensitivity and specificity in a screening situation, sensitivity and specificity can be useful in two circumstances but *only if* they are extremely high. First, because a highly sensitive screening test is unlikely to produce false negative outcomes (there will be few entries in cell c of Figure 1), people who test negative on that kind of screening test (i.e., a test with high sensitivity) are very unlikely to have the target condition. Expressed differently, high sensitivity permits people to be confidently regarded as not having a condition if their screening test yields a negative result. They can be “ruled out.” This has led to the mnemonic *snout* (sensitive, negative, out—in which it is useful to regard the *n* in *snout* as referring to the *n* in *sensitive* as well as the *n* in *negative*) concerning high sensitivity in screening.

Second, because a highly specific screening test is unlikely to produce false positive results (there will be few entries in cell b in Figure 1), people are very unlikely to be categorized as having a condition if they indeed do not have it. Expressed differently, high specificity permits people to be confidently regarded as having a condition if their diagnostic test yields a positive result. They can be “ruled in”—and, thus, the mnemonic *spin* (specific, positive, in—in which it is useful to regard the *p* in *spin* as referring to the *p* in *specific* as well as the *p* in *positive*) concerning high specificity in screening.

The mnemonics *snout* and *spin*, it must be emphasized, pertain only when sensitivity and specificity are high. Their pliability, therefore, has some strong limitations. Furthermore, these mnemonics are applied in a way that might seem counterintuitive. A screening test with high sensitivity is not necessarily useful for “picking things up.” It is useful *only* for deciding that a negative screening test outcome is so unusual that it strongly indicates the *absence* of the target condition. Conversely, a screening test with high specificity is not so “choosy” that it is effective in ignoring a condition if that condition is not present; rather, a highly specific test is useful *only* for deciding that a positive screening test outcome is so unusual that it strongly indicates the *presence* of the target condition. In addition, Pewsner et al. (2) have pointed out that effective use of snout and spin is “eroded” when highly sensitive tests are not sufficiently specific or highly specific tests are not sufficiently sensitive—and for many screening tests, unfortunately, either sensitivity or specificity is low despite the other being high, or neither sensitivity nor specificity is high. As a consequence, both sensitivity and specificity remain unhelpful for making decisions about individual people in most screening contexts, and PPV and NPV should be retained as the metrics of choice in those contexts.

## Assessing Desirable Predictive Values and Consequences for Sensitivity and Specificity

When assessing the desirability of specific PPVs and NPVs, a variety of costs and benefits need to be considered (1). These include the immediate and long-term burdens on the healthcare system, the treatability of a particular condition, and the psychological effect on clients as well as clients’ health status. Considerations might also include over- versus under-application of diagnostic procedures as well as the possibility of premature versus inappropriately delayed application of diagnostic procedures. Input from clinicians and policymakers is likely to be particularly informative in any deliberations.

Decisions about desirable PPVs and NPVs can be approached from two related and complementary, but different, directions. One approach involves the extent to which true positive and true negative results are desirable on a screening test. The other approach involves the extent to which false positive and false negative results are tolerable or even acceptable.

A high PPV is desirable, implying that false positive outcomes are minimized, under a variety of circumstances. Some of these are when, relative to potential benefits, the costs (including costs associated with finances, time, and personnel for health services, as well as inconvenience, discomfort, and anxiety for clients) are high. A high PPV, with its concomitant few false positive screening test results, is also desirable when the risk of harm from follow-up diagnosis or therapy (including hemorrhaging and infection) is high despite the benefits from treatment also being high, or when the target condition is not life-threatening or progresses slowly. Under these circumstances, false positive outcomes can be associated with overtreatment and unnecessary costs and prospect of iatrogenic complications. False positive outcomes may also be annoying and distressing for both the providers and the recipients of health care.

A moderate PPV (with its greater proportion of false positive screening test outcomes) might be acceptable under a number of circumstances, most of which are the opposite of the situations in which a high PPV is desirable. For example, a certain percentage of false positive outcomes might not be objectionable if follow-up tests are inexpensive, easily and quickly performed, and not stressful for clients. In addition, false positive screening outcomes might be quite permissible if no harm is likely to be done to clients in protecting them against a target condition even if that condition is not present. For example, people who are mistakenly told that they have peripheral artery disease, despite not actually having it, are likely to benefit from adopting advice to exercise appropriately, improve their diet, and discontinue smoking.

A high NPV is desirable, implying that false negatives are minimized, under a different set of circumstances. Some of these are a condition being serious, largely asymptomatic, or contagious, or if treatment for a condition is advisable early in its course, particularly if the condition can be treated effectively and is likely to progress quickly. Under these circumstances, it would be highly undesirable if a screening test indicated that people did not have a condition when in fact they did. A moderate NPV—with its greater proportion of false negative screening test outcomes—might be acceptable under other circumstances, however, and most of those circumstances are the opposite of those that make a high NPV desirable. For example, the false negative outcomes associated with moderate NPVs might not be problematic if the target condition is not serious or contagious, or if a condition does not progress quickly or benefit from early treatment. Moderate NPVs might also be acceptable if diagnosis at low levels of a condition is known to be ambiguous and subsequent screening tests can easily be scheduled and performed, or if, given time, a condition is likely to resolve itself satisfactorily without treatment.

If, for a variety of reasons, the PPVs and NPVs on a screening test were deemed to be either too high or too low, they could be adjusted by altering the stringency of the screening test (for example, by raising or lowering cutpoints on a continuous variable or by changing the components that comprise a screening test), by altering the sample of people on whom the analyses were based (for example, by identifying people who are regarded as having more pertinent demographic or health status variables), or by altering the nature of the reference standard. Those strategies would almost inevitably result in changes to the sensitivity and specificity values, and those revised values would simply need to be reported as applying to the particular new level of stringency on the screening test, the applicable population, and the reference standard when that test was being described. This reveals, yet again, that pliability can be associated with sensitivity, specificity, and predictive values.

## The Importance of Full Disclosure of Information in Research

When describing screening tests, many researchers provide information about their reference standard; the prevalence of the target condition in their research sample(s); the criteria that had been used to indicate presence or absence of a condition according to the screening test; and the sensitivity, specificity, and predictive values they obtained (6, 7, 15, 17, 18). The research results are not always impressive or what the researchers might have hoped for, but at least it is possible to draw informed conclusions from those results.

Sometimes only partial information is provided, and that limits the usefulness of research. For example, in a systematic review concerning the toe–brachial index in screening for peripheral artery disease, Tehan et al. (19) were evidently unable to find predictive values in so many of the final seven studies they reviewed that they did not provide any information about those values—despite those metrics being of fundamental importance for screening.

In one of the more informative articles reviewed by Tehan et al. (19), Okamoto et al. (13) did include information about sensitivity, specificity, and predictive values of several screening tests. However, they provided insufficient interpretation at times. For example, they reported an unusually low sensitivity value of 45.2% for the toe–brachial index in detecting peripheral artery disease. That value occurred in the presence of 100% specificity, indicating that the cutoff point might have been too stringent and that sensitivity had been sacrificed in the interest of obtaining high specificity, but the researchers did not draw attention to that or provide any explanation for their strategy. Information in a receiver operating characteristic analysis within their article suggests that more appropriate sensitivity and specificity values would have both been approximately 73% and therefore, incidentally, similar to the values obtained by other researchers (15, 20).

Deficiencies in provision of information can be even more problematic. In a recently published article, Jönelid et al. (21) investigated usefulness of the ankle–brachial index for identifying polyvascular disease. Although they reported a specificity of 92.4% and a PPV of 68.4%, they did not provide results concerning either sensitivity or NPV. From information in their article, those unrevealed values can be calculated as both being 100%. That these values are so high in a screening context raises suspicions. When following those suspicions through, it becomes evident that the researchers used the ABI as a component of the reference standard *as well as* being the sole variable that comprised the screening test. Failure to sufficiently disclose this circular situation (the inevitability of something being highly related to something that is partly itself) permitted the authors to claim that the “ABI is a useful … measurement that appears predictive of widespread atherosclerosis” in their patients. That this statement is invalid becomes apparent only through an awareness of how researchers’ data should conform to entries in Figure 1 and how reference standards and screening tests are conceptualized.

The above examples illustrate the importance of research consumers being provided with complete information when screening tests are being described, and consumers being able to interpret that information appropriately—sometimes with at least a modicum of skepticism. Having a healthy level of skepticism as well as clarity concerning the nature and appropriate interpretations and uses of sensitivity, specificity, and predictive values, can be seen as important for educators, researchers, and clinicians in public health.

## Summary

- Sensitivity and specificity should be emphasized as having different origins, and different purposes, from PPVs and NPVs, and all four metrics should be regarded as important when describing and assessing a screening test’s adequacy and usefulness.
- Researchers and clinicians should avoid confusion of the inverse when considering the application of sensitivity and specificity to screening tests.
- Predictive values are more relevant than are sensitivity and specificity when people are being screened.
- Predictive values on screening tests need to be determined on the basis of careful clinical deliberation and might be used in a reverse process that would result in adjustments to sensitivity and specificity values.
- Researchers should provide information about sensitivity, specificity, and predictive values when describing screening test results, and that information should include how those metrics were derived as well as appropriate interpretations.

## Author Contributions

RT conceived of, conducted the research for, and wrote the complete manuscript.

## Conflict of Interest Statement

The author declares that this research was conducted in the absence of any commercial or financial relationships that could be construed as a potential conflict of interest.
